# Gate Tunable Transport in Graphene/MoS_2_/(Cr/Au) Vertical Field-Effect Transistors

**DOI:** 10.3390/nano8010014

**Published:** 2017-12-28

**Authors:** Ghazanfar Nazir, Muhammad Farooq Khan, Sikandar Aftab, Amir Muhammad Afzal, Ghulam Dastgeer, Malik Abdul Rehman, Yongho Seo, Jonghwa Eom

**Affiliations:** 1 Department of Physics & Astronomy and Graphene Research Institute, Sejong University, Seoul 05006, Korea; zafarforall2004@gmail.com (G.N.); muhammadfarooqkhan87@gmail.com (M.F.K.); physics.sikandar@gmail.com (S.A.); Amirafzal461@gmail.com (A.M.A.); dtedastgeer@gmail.com (G.D.); 2 Department of Nanotechnology & Advanced Materials Engineering, Sejong University, Seoul 05006, Korea; malik.mann002@gmail.com (M.A.R.); yseo@sejong.ac.kr (Y.S.)

**Keywords:** vertical transport, transition metal dichalcogenides, MoS_2_, graphene

## Abstract

Two-dimensional materials based vertical field-effect transistors have been widely studied due to their useful applications in industry. In the present study, we fabricate graphene/MoS_2_/(Cr/Au) vertical transistor based on the mechanical exfoliation and dry transfer method. Since the bottom electrode was made of monolayer graphene (Gr), the electrical transport in our Gr/MoS_2_/(Cr/Au) vertical transistors can be significantly modified by using back-gate voltage. Schottky barrier height at the interface between Gr and MoS_2_ can be modified by back-gate voltage and the current bias. Vertical resistance (R_vert_) of a Gr/MoS_2_/(Cr/Au) transistor is compared with planar resistance (R_planar_) of a conventional lateral MoS_2_ field-effect transistor. We have also studied electrical properties for various thicknesses of MoS_2_ channels in both vertical and lateral transistors. As the thickness of MoS_2_ increases, R_vert_ increases, but R_planar_ decreases. The increase of R_vert_ in the thicker MoS_2_ film is attributed to the interlayer resistance in the vertical direction. However, R_planar_ shows a lower value for a thicker MoS_2_ film because of an excess of charge carriers available in upper layers connected directly to source/drain contacts that limits the conduction through layers closed to source/drain electrodes. Hence, interlayer resistance associated with these layers contributes to planer resistance in contrast to vertical devices in which all layers contribute interlayer resistance.

## 1. Introduction

Heterostructures [1,2,3] composed of graphene and other two-dimensional (2D) crystals, such as transition metal dichalcogenides (TMDs), are of a great interest due to their fundamental and applied aspects. Extensive research has been carried out in both lateral [4,5,6] and vertical [3,7,8,9,10,11] hetero-stacks of graphene with other two-dimensional materials. The vertical devices of two-dimensional materials open particularly promising new horizons in material research. For example, graphene vertical field-effect transistors (G-VFETs) are charming candidates for future research, as they have ultimately thin bodies of a few atomic layers, which provide ultrafast transport nearly in a few femtoseconds [12] and higher switching (on/off) ratio as compared to their lateral counterparts.

Thus far, several vertical heterostructures of graphene with other 2D materials have been proposed. For example, Britnell et al. introduced a VFET heterostructure [13] composed of two graphene electrodes and a thin hexagonal boron nitride (h-BN) layer sandwiched between them showing the on/off ratio ~50. When h-BN was replaced by MoS_2_, on/off ratio was enhanced to ~10^4^ due to the low bandgap of MoS_2_ as compared to h-BN. It was reported that tunneling transport was dominant in off-state, whereas thermionic transport played a major role in on-state in other previous studies on Gr/TMDs/Gr vertical devices [3]. Another configuration of VFETs has been investigated in which one side of TMDs was contacted with a graphene electrode, while the other was contacted with a metal electrode [7,14,15,16,17,18,19,20,21]. In this kind of devices (Gr/TMDs/Metal), Schottky barriers at the interface between graphene and TMDs play an important role in the electrical transport. Electric field from the back-gate can modify the Schottky barrier height. These devices are superior to tunneling devices due to a large current density through the semiconducting TMDs channel [22,23,24]. Gr/TMDs/Metal devices showed a high on/off ratio and low driving voltage; therefore, they are good to use in low power consumption applications.

In previous reports, most VFETs were studied by employing the two-probe measurement configuration, which included contact electrodes as a part of the devices. This configuration always possesses contact resistance due to which intrinsic characteristics of the device were impossible to achieve. In the present paper, we have fabricated Gr/MoS_2_/(Cr/Au) VFET with various MoS_2_ thickness. However, we use four-probe cross-bar geometry in this experiment to exclude contribution of electrode to the measurement. Monolayer Gr used as a bottom electrode allows back-gate electric field to tune the energy states of MoS_2_ in VFET. Therefore, we could investigate the gate-dependent electrical transport in our MoS_2_ VFET. Moreover, graphene contacts [25,26,27,28] can effectively decrease contact resistance because of small work function mismatch between graphene and MoS_2_. We have also measured planar transport properties of lateral MoS_2_ field-effect transistors for comparison. Electronic transports in the vertical and lateral direction were discussed by analyzing the resistance components. 

## 2. Experimental Section

### 2.1. Device Fabrication

Our Gr/MoS_2_/(Cr/Au) VFETs were fabricated on SiO_2_/P^+^-Si substrate. The thickness of dielectric (SiO_2_) was 300 nm, whereas P^+^-Si was used to apply back-gate voltage. In the first process, chemical vapor deposition (CVD) grown graphene was transferred on SiO_2_/Si substrate using wet transfer method [29], and a graphene Hall bar pattern was defined by using photo-lithography. Then oxygen plasma (power ~50 W) was used for several minutes to etch extraneous graphene. The Raman spectrum for Gr is shown in Supplementary Materials Figure S1 (see Supplementary Materials). In Raman spectrum, 2D to G peak intensity ratio is larger than 3:1, indicating monolayer characteristics of Gr [30]. We used the scotch-tape method for mechanical exfoliation of MoS_2_ [17,31,32,33,34,35,36,37,38,39,40,41,42,43,44,45]. The suitable MoS_2_ flake was selected under an optical microscope and then transferred on graphene Hall bar by using micromanipulator. Subsequently, 15-nm-thick HfO_2_ films was grown by atomic layer deposition on the e-beam lithography defined area. In the last process, e-beam lithography was done to define the top electrode of Cr/Au (8/120 nm).

### 2.2. Measurements

Electrical measurements were performed by using Keithley 2400 source meter, Keithley 6485K picoammeter, and Keithley 2182A nanovoltmeter. All measurements were performed in vacuum at room temperature. Structural investigation and material identification were performed using Raman spectroscopy and atomic force microscopy (AFM). In Raman spectroscopy, laser wavelength of 514 nm with power below 1 mW was selected to avoid structural degradation caused by the heating effects of the laser. The nominal diameter of laser spot was 0.7 µm. 

## 3. Results and Discussion

### 3.1. Gr/MoS_2_/(Cr/Au) Vertical Field-Effect Transistor

**Figure 1:**
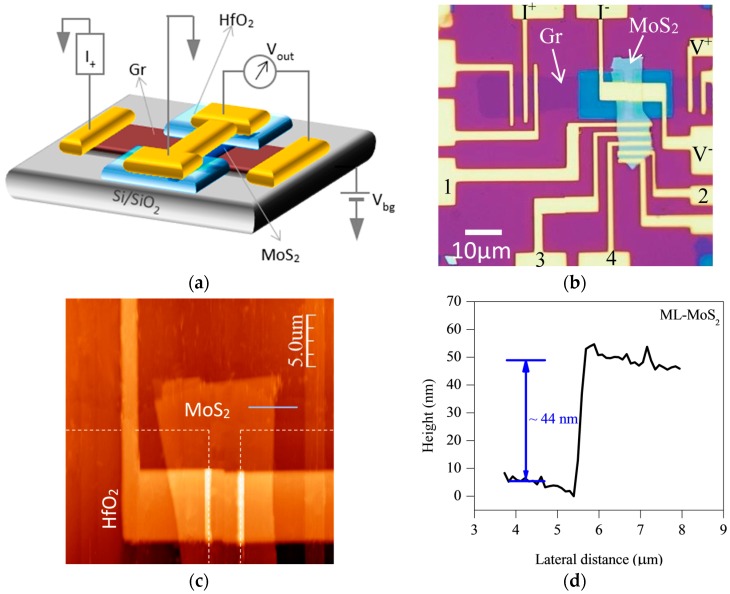
(**a**) Schematic representation of Gr/MoS_2_/(Cr/Au) vertical field-effect transistor (VFET) (**b**) Optical image of Gr/MoS_2_/(Cr/Au) VFET. Two HfO_2_ windows on top of Gr/MoS_2_ layer define the junction region. Different contacts with alphabetic and numeric letters were used to measure R_vert_ and R_planer_, respectively (**c**) Atomic force microscope image that clearly reveals multilayer MoS_2_ (ML-MoS_2_) flake with top Cr/Au contact. HfO_2_ windows are highlighted by dashed lines. (**d**) Height profile of ML-MoS_2_ shows the thickness of nearly 44 nm.

A schematic representation of Gr/MoS_2_/(Cr/Au) VFET is shown in Figure 1a, where the back-gate voltage is applied to control the vertical transport in MoS_2_ channel. The optical micrograph of device is shown in Figure 1b. Graphene is represented by the purple color, MoS_2_ by the sky blue, and HfO_2_ (~15 nm thick) window by the dark blue color. We choose CVD-grown monolayer graphene despite multilayer graphene. We believe that, in case of multilayer graphene, the gate effect would be small as compared to monolayer graphene due to back-gate electric field screening, and modulation in Schottky barrier height will be more difficult as compared to monolayer graphene.

The alphabetic and numeric symbols indicate different contacts used for the planar and vertical transport measurement. Figure 1c shows AFM of MoS_2_ film, which reveals uniform surface morphology. Height profile taken by AFM shows the thickness of MoS_2_ to be ~44 nm in Figure 1d. 

Raman spectroscopy of MoS_2_ on monolayer Gr was studied in comparison with MoS_2_ on Si/SiO_2_ substrate shown in Supplementary Materials Figure S2. Raman spectrum for MoS_2_ on Si/SiO_2_ substrate was represented by the black color, whereas the red color shows Raman spectrum of MoS_2_ on Gr. Two prominent peaks of MoS_2_ appeared in the wavenumber range from 380 to 420 cm^−1^. These two Raman peaks belongs to in-plane ($E_{2g}^{1}$) and out of plane (A_1g_) vibrations of “Mo” and “S” atoms [46]. The difference between $E_{2g}^{1}$ and A_1g_ Raman peaks for MoS_2_ on Si/SiO_2_ substrate amounted to Δ ≈ 24 cm^−1^, indicating a multilayer nature [22]. There was a slight change in peak positions of MoS_2_ when stacked on Gr. This slight change was due to the relaxation of atoms on different substrate.

We assume that vertical resistance (R_vert_) is a cumulative effect of all the resistances including contact resistance and MoS_2_ channel resistance. We made a cross-junction geometry to investigate the vertical transport in Gr/MoS_2_/(Cr/Au). The measurements consisted of four-probe technique where two contacts were used as source and drain, while the other two for voltage measurement across VFET. Figure 1b shows the measurement configuration (I^+^, I^−^, V^+^, V^−^) for the vertical resistance R_vert_. We also measured planar resistance R_planar_ of the same MoS_2_ film using Cr/Au contacts as illustrated by numeric symbols 1, 2, 3, 4 in Figure 1b.

### 3.2. Vertical Resistance of Gr/MoS_2_/(Cr/Au) VFET

**Figure 2:**
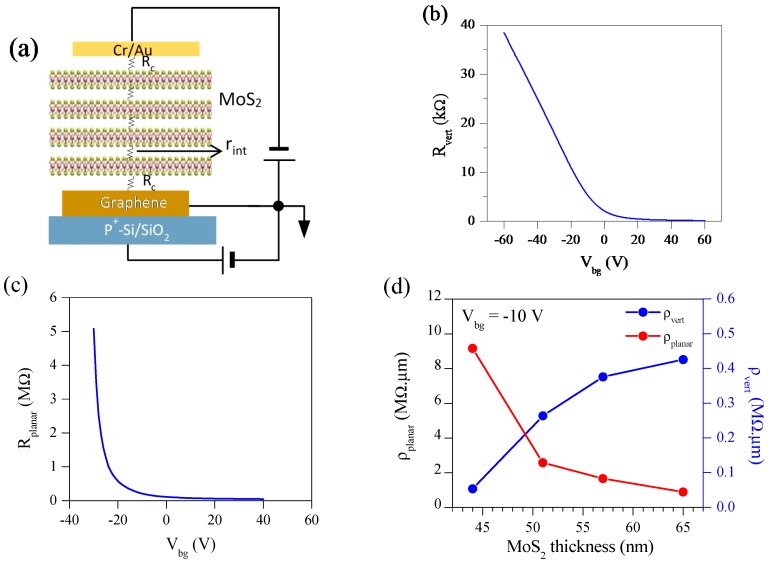
(**a**) Schematic diagram for resistances to compose Gr/MoS_2_/(Cr/Au) vertical field-effect transistor (VFET) with 50 nm-thick MoS_2_. (**b**) Vertical Resistance (R_vert_) as a function of the back-gate voltage (V_bg_) for Gr/MoS_2_/(Cr/Au) VFET with 50 nm-thick MoS_2_. (**c**) Planar resistance (R_planar_) as a function of V_bg_ for the lateral MoS_2_ field-effect transistor with 48 nm-thick MoS_2_. (**d**) Dependence of $\rho_{\mathrm{planar}}$ and $\rho_{\mathrm{vert}}$ on the thickness of MoS_2_ channels at V_bg_ = −10 V.

Figure 2a shows a schematic diagram of Gr/MoS_2_/(Cr/Au) VFET, where R_int_ and R_c_ represent interlayer resistance between stacked layer of MoS_2_ and contact resistance, respectively. Figure 2b represents vertical resistance (R_vert_) of Gr/MoS_2_/(Cr/Au) VFET as a function of the back-gate voltage (V_bg_). In this experiment, the current flows from Gr to the top Cr/Au contact through semiconducting MoS_2_ channel. The electrical characteristics are strongly modified by V_bg_. To understand the physics of the transport mechanism, there are lot of factors that should be keep in mind e.g., Schottky barrier height, barrier width etc. It is a well-known concept that electrical transport in TMD’s based field effect transistors (FET) is governed by either tunneling or thermionic emission. Tunneling is due to passing of carriers through barrier height and is a temperature-independent quantity. However, thermionic emission is highly reliant on temperature. Tunneling current depends upon barrier width, and if the barrier width is too large, we cannot observe tunneling current. Here, our average MoS_2_ thickness is 50 nm (barrier width ~50 nm). Usually, to observe tunneling mechanism, the barrier width should be ≤5 nm [47]. So, we can firmly say that in our experiment, electrical transport is not caused by tunneling. However, the low value of current observed at V_bg_ < 0 in our Gr/MoS_2_ heterostructure’s transport property (shown in Supplementary Materials Figure S3c) is due to gate leakage current and not by the tunneling that is a very well-known phenomenon in Si-based field effect transistors. The Gr/MoS_2_ junction is completely in off state at low V_bg_ due to the presence of large Schottky barrier height (SBH), which is why we observed large resistance at that point.

On the other hand, Deshun Qu et al. reported that “S” vacancies in MoS_2_ play an important role in r_int_ [48]. The importance of “S” atoms was further elaborated for the vertical carrier transport by orbital overlapping between “S” atoms in adjacent layers [49,50].

### 3.3. Resistance Analysis of Gr/MoS_2_/(Cr/Au) VFET

R_vert_ in the measurement configuration of Figure 2a can be considered as the sum of interface resistance between Gr and MoS_2_ ($R_{\mathrm{Gr}/{\mathrm{MoS}}_{2}}$), total channel resistance of individual layers of MoS_2_ (R_TCR-V_), interlayer resistance (R_int_) of MoS_2_, and interface resistance (R_SBH_) between Cr/Au electrode and MoS_2_. So, the vertical resistance (R_vert_) is given by Equation (1).

(1) R_vert_ = R_TCR__-V_ + R_int_ + R_GR/MoS_2__ + R_SBH_

where total interlayer resistance (R_int_) is given by $R_{\mathrm{int}}{=\mathrm{Nr}}_{\mathrm{int}}$, where N is the total number of layers in MoS_2_ and r_int_ is the interlayer resistance between two consecutive layers of MoS_2_. As the thickness of MoS_2_ increases, R_int_ increases, and then R_vert_ increases as well. In the vertical transport, r_int_ is a non-negligible quantity in the thick MoS_2_ channel. Another important factor that should be elaborated here is the channel resistance itself. In the vertical transport, R_TCR-V_ increases with an increase of the thickness of MoS_2_ channel. Since both R_int_ and R_TCR-V_ increase with increasing MoS_2_ thickness, R_vert_ shows the dependence of MoS_2_ thickness. However, the resistance of planar or vertical device depends on cross-sectional area, so it is better to examine resistivity instead of resistance. Figure 2d shows $\rho_{\mathrm{planar}}$ and $\rho_{\mathrm{vert}}$ as a function of MoS_2_ thickness.

**Figure 3:**
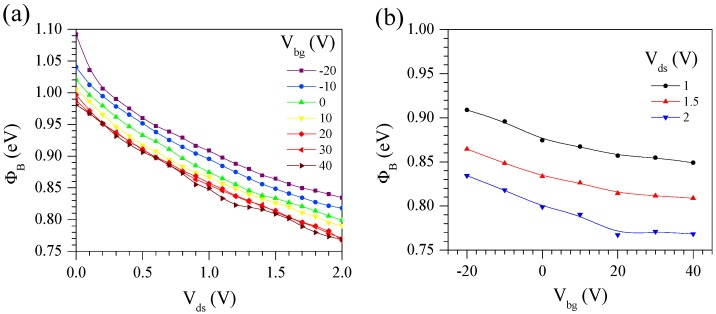
(**a**) Schottky barrier height (ϕ_B_) at T = 300 K between monolayer Gr and 53-nm-thick ML-MoS_2_ as a function of V_ds_ at different V_bg_’s from −20 to 40 V with equal step of 10 V. (**b**) ϕ_B_ at T = 300 K as a function of V_bg_ at V_ds_ = 1, 1.5, and 2 V.

As another important component of R_vert_, we discuss $R_{\mathrm{Gr}/{\mathrm{MoS}}_{2}}$ which is the interface resistance between Gr and MoS_2_. We analyze $R_{\mathrm{Gr}/{\mathrm{MoS}}_{2}}$ within 2D thermionic emission theory, where the Schottky barrier height is given by Equation (2).

(2) $$\Phi_{B}=\frac{k_{B}T}{e}\ln(\frac{A^{*}T^{2}}{J_{\mathrm{rev}}})$$

where $k_{B}$ is the Boltzmann’s constant, A* is effective Richardson constant and we choose A* = 54 Acm^−2^ K^−2^ for MoS_2_ [51]. We extracted Schottky barrier height at T = 300 K from I_ds_-V_ds_ curves shown in Supplementary Materials Figure S3. J_rev_ (=I_ds_/A_junc_) is the reverse saturation current density at V_ds_ = −0.5 V, where we see a beginning of saturation of I_ds_. A_junc_ (=200 μm^2^) is the junction area of Gr/MoS_2_. Barrier height decreases with an increase of V_ds_ or V_bg_ (see Figure 3a,b). $\Phi_{B}$ ranges from 1.10 to 0.78 eV for the interface between monolayer Gr and 53-nm-thick ML-MoS_2_.

Moreover, planar resistance (R_planar_) of the MoS_2_ channel was measured by using the electrodes labelled as numeric letters in Figure 1b. While current was applied between 1 and 2, voltage was measured between 3 and 4. Figure 2c shows R_planar_ as a function of V_bg_. As V_bg_ increases from −30 to 40 V, R_planar_ rapidly decreases, indicating MoS_2_ is a *n*-type semiconductor. The MoS_2_ channel thickness dependence of R_planar_ at V_bg_ = −10 V is shown in Figure 2d. As MoS_2_ flake thickness increases, R_planar_ decreases in contrast to R_vert._ We can analyze R_planar_ as R_TCR-P_ in 4-probe measurement configuration. Here, R_TCR-P_ represents the in-plane resistance of MoS_2_. As the thickness of MoS_2_ channel increases, more layers can contribute to the planar electrical transport, so that total planer channel resistance, R_TCR-P_ decreases. To have an estimate of contact resistivity, we employed transmission line method (TLM) on 51 nm-thick MoS_2_ flake and extracted contact resistivity to be 0.14 MΩ μm at V_bg_ = −10 V as shown in Supplementary Materials Figure S4b. The contact resistivity is smaller than the planar resistivity of MoS_2_ channel itself. So, it is obvious that there are other factors contributing to the total in-plane resistance other than contacts. The back-gate voltage-dependent contact resistivity was also estimated using TLM method as shown in Supplementary Materials Figure S4d. The contact resistivity decreases with V_bg_ and has a low value in the positive region of V_bg_ due to the increasing carrier channels.

In lateral devices, the source and drain contacts are directly attached to top surface of MoS_2_. Therefore, the charge carriers will flow from source to drain mainly through a few top layers of MoS_2_ due to the interlayer resistance [52,53]. Moreover, since the thickness of MoS_2_ flake in the lateral device is ~50 nm (about 76 layers), the back-gate electric field will not affect much on the charge carrier transport. This could be the reason of a low R_planer_ for thick MoS_2_ channel in the lateral devices.

### 3.4. Vertical Transport of (Cr/Au)/Mos_2_/(Cr/Au) Vertical Field-Effect Transistor

**Figure 4:**
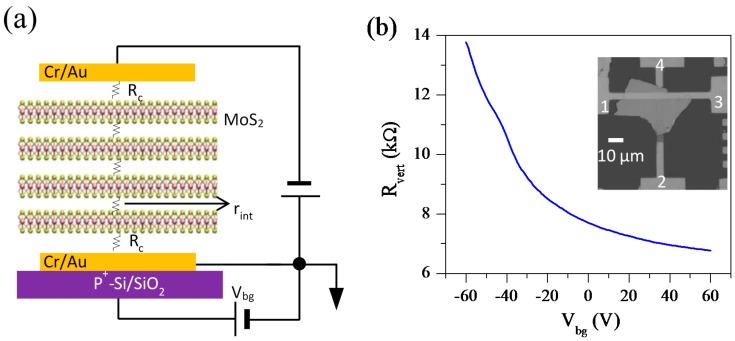

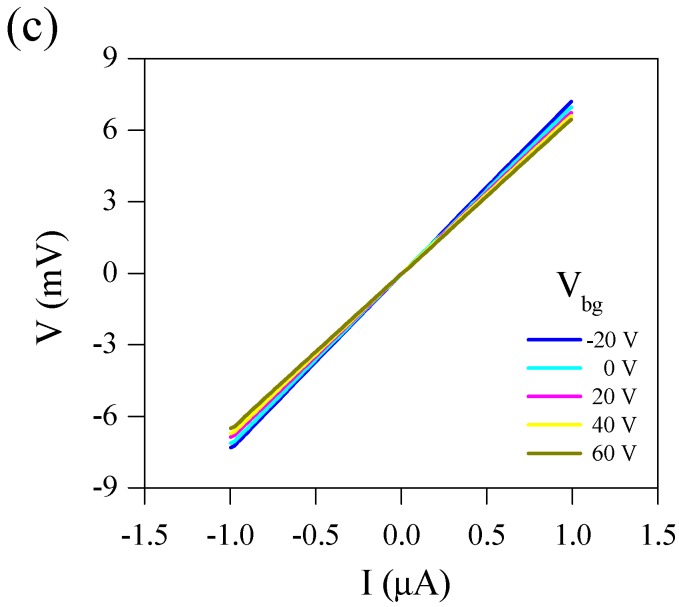
(**a**) Schematic diagram of the resistances to compose (Cr/Au)/MoS_2_/(Cr/Au) vertical field-effect transistor (VFET). The thickness of MoS_2_ is 48 nm. (**b**) R_vert_ as a function of V_bg_ for (Cr/Au)/MoS_2_/(Cr/Au) VEFT. Inset: A scanning electron microscope image of the device. While the constant current of 1 μA is applied between 1 and 4, voltage is measured between 2 and 3. (**c**) I-V characteristics at different V_bg_’s.

We have studied vertical transport in (Cr/Au)/MoS_2_/(Cr/Au) VFET, the schematic representation of which is shown in Figure 4a, and inset of Figure 4b shows the scanning electron microscope image. The thickness of MoS_2_ is about 48 nm. While current bias is applied between 4 and 3, voltage is measured between 2 and 1. Figure 4b shows R_vert_ as a function of V_bg_ at room temperature. It shows similar characteristics as Gr/MoS_2_/(Cr/Au) VFET, but with a low R_vert_, which may be due to the low interface resistance between Cr/Au and MoS_2_. The strong screening of electric field by the bottom Cr/Au electrode creates a weaker dependence on V_bg_. The I-V characteristics show almost ohmic behavior at different back-gate voltages (see Figure 4c).

**Figure 5:**
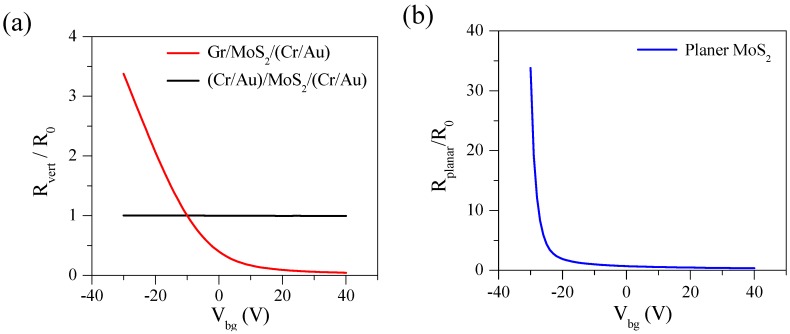
(**a**) R_vert_/R_0_ as a function of V_bg_. While the thickness of MoS_2_ is 50 nm for Gr/MoS_2_/(Cr/Au) VEFT, the thickness of MoS_2_ is 48 nm for (Cr/Au)/MoS_2_/(Cr/Au) VEFT. (**b**) R_planar_/R_0_ as a function of V_bg_ for 48 nm-thick MoS_2_ in planar geometry. Reference resistances (R_0_) were taken at V_bg_ = −10 V. All measurements were done at T = 300 K.

Although R_vert_ of (Cr/Au)/MoS_2_/(Cr/Au) VFET in Figure 4b decreases as V_bg_ is increased, the relative change of R_vert_ is rather small. To compare the relative change of R_vert_ we obtain R_vert_/R_0_ as a function of V_bg_, where R_0_ at V_bg_ = −10 V is taken as a reference resistance. Figure 5a shows R_vert_/R_0_ as a function of V_bg_ for Gr/MoS_2_/(Cr/Au) and (Cr/Au)/MoS_2_/(Cr/Au) VFETs. The thickness of MoS_2_ is 50 nm and 48 nm for Gr/MoS_2_/(Cr/Au) and (Cr/Au)/MoS_2_/(Cr/Au) VFETs, respectively. Of note, V_bg_ does not significantly affect transport characteristics in (Cr/Au)/MoS_2_/(Cr/Au) VFET, because the bottom Cr/Au electrode effectively screens the electric field from the V_bg_ application. However, R_vert_/R_0_ has a strong dependence on V_bg_ in Gr/MoS_2_/(Cr/Au) devices, due to the weak screening of the electric field by bottom monolayer Gr. For comparison, we also present R_planar_/R_0_ as a function of V_bg_ in Figure 5b. A rapid increase of R_planar_/R_0_ below V_bg_ = −30 V indicates the threshold voltage of *n*-type MoS_2_ channel in our devices. The dependence of R_planar_/R_0_ on V_bg_ is much larger than VFETs, asMoS_2_ channel is directly affected by V_bg_ application in ordinary planar geometry.

In the positive region of V_bg_, both resistances in planar (in-plane) and vertical configuration show a similar dependence of V_bg_. However, at negative V_bg_, we find different dependences of V_bg_ as seen in Figure 5a,b. In Figure 5a R_vert_/R_0_ shows an exponential dependence of V_bg_ for Gr/MoS_2_/(Cr/Au) VFET with 50 nm-thick MoS_2_ flake. This might be due to the gate-voltage dependent density of states in graphene, which affects the interface resistance between graphene and MoS_2_. In case of (Cr/Au)/MoS_2_/(Cr/Au) VFET with 50 nm-thick MoS_2_, there exists large screening effect by the bottom Cr/Au electrode that does not allow the back-gate electric field to influence on the electronic transport. On the other hand, in case of planar 48 nm-thick MoS_2_ FET of Figure 5b, we have source/drain (Cr/Au) electrodes directly on the lateral channel of MoS_2_. At a particular negative V_bg_, the channel transport is in off-state, so resistance increases rapidly at V_bg_ below the threshold voltage.

## 4. Conclusions

In the present study, we performed four probe measurements using cross-junction geometry of Gr/MoS_2_/(Cr/Au) VFETs. We were able to achieve gate-tunable transport characteristics in VFETs. Since the bottom Gr allows the electric field to reach MoS_2_ channel, the vertical resistance (R_vert_) of Gr/MoS_2_/(Cr/Au) VFET can be effectively modified by V_bg_. The vertical transport characteristics are examined as compared to those of the ordinary lateral MoS_2_ field-effect transistor. R_vert_ increases as the thickness of MoS_2_ increases, whereas R_planar_ decreases. The increase of R_vert_ in the thicker MoS_2_ film can be attributed to the interlayer resistance in the vertical direction. However, R_planar_ shows a lower value for a thicker MoS_2_ film because of excess of charge carriers available in upper layers connected directly to source/drain contacts and cumulative contribution of low R_int_ from the lower layers. In planer geometry only, layers attached near to channel layer (on which source/drain electrodes are constructed) are the main source of the conduction mechanism. Schottky barrier height at Gr/MoS_2_ interface can be analyzed from the I_ds_-V_ds_ curve. The Schottky barrier height decreases as V_bg_ or V_ds_ increases. V_bg_ does not affect transport characteristics much in (Cr/Au)/MoS_2_/(Cr/Au) VFET, because the bottom Cr/Au electrode effectively screens the electric field from the V_bg_ application. However, a strong dependence on V_bg_ in Gr/MoS_2_/(Cr/Au) devices was observed due to the weak screening of electric field by bottom Gr. We believe that gate tunable VFET will serve as one of important components for future 2D materials electronics.

## Supplementary Materials

The following are available online at www.mdpi.com/2079-4991/8/1/14/s1.
