# Supplementary material for: Gate Tunable Transport in Graphene/MoS2/(Cr/Au) Vertical Field-Effect Transistors
Source: Nanomaterials (Basel). 2017 Dec 28;8(1):14. doi: 10.3390/nano8010014 (PMC5791101; doi:10.3390/nano8010014)
Supplement: Supplementary file 1 [file nanomaterials-08-00014-s001.pdf]

Supplementary Materials

Gate Tunable Transport in Graphene/MoS_2/(Cr/Au) Vertical Field-Effect Transistors

Ghazanfar Nazir [1], Muhammad Farooq Khan [1], Sikandar Aftab [1], Amir Muhammad Afzal [1], Ghulam Dastgeer [1], Malik Abdul Rehman [2], Yongho Seo [2], and Jonghwa Eom [1,*]

[1] Department of Physics & Astronomy and Graphene Research Institute, Sejong University, Seoul 05006, Korea; zafarforall2004@gmail.com (G.N.); muhammadfarooqkhan87@gmail.com (M.F.K.); physics.sikandar@gmail.com (S.A.); Amirafzal461@gmail.com (A.M.A.); dtedastgeer@gmail.com (G.D.)

[2] Department of Nanotechnology & Advanced Materials Engineering, Sejong University, Seoul 05006, Korea; malik.mann002@gmail.com (M.A.R.); yseo@sejong.ac.kr (Y.S.)

* Correspondence: eom@sejong.ac.kr

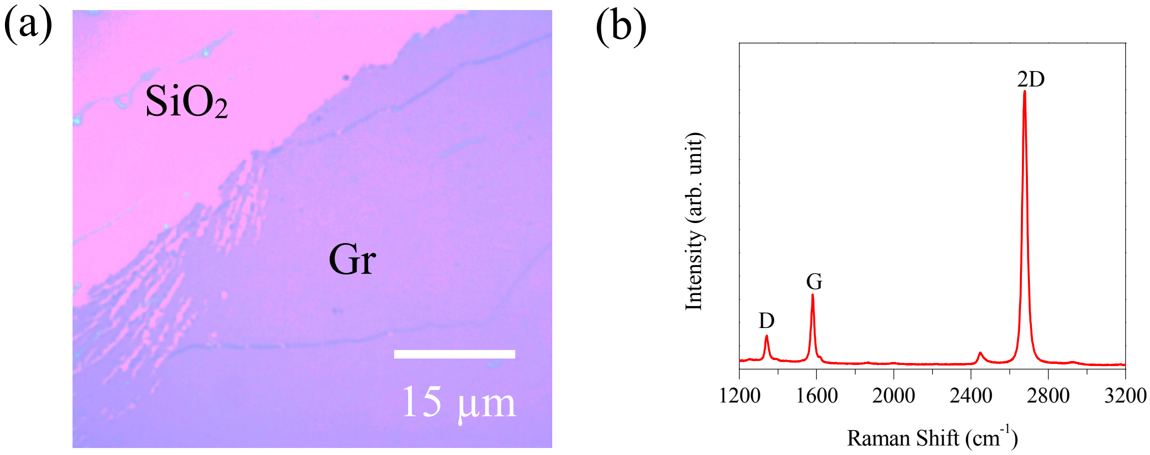

Figure S1. (a) An optical microscope image of monolayer graphene. (b) Raman spectra of monolayer graphene. Small D peak indicates fewer defects. The ratio of $I_{2D}/I_G > 2$ indicates that our graphene is monolayer.

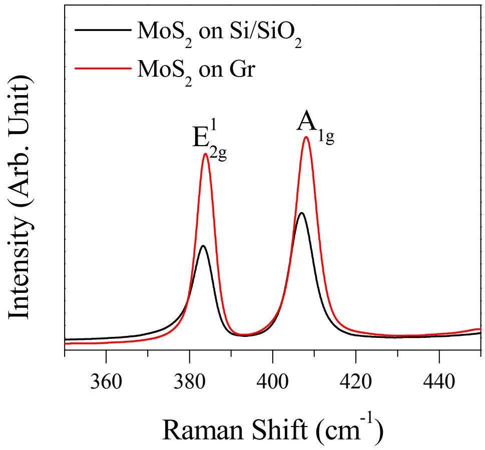

Figure S2. Raman spectra of MoS_2 on Si/SiO_2 substrate (black curve) and on graphene (red curve)

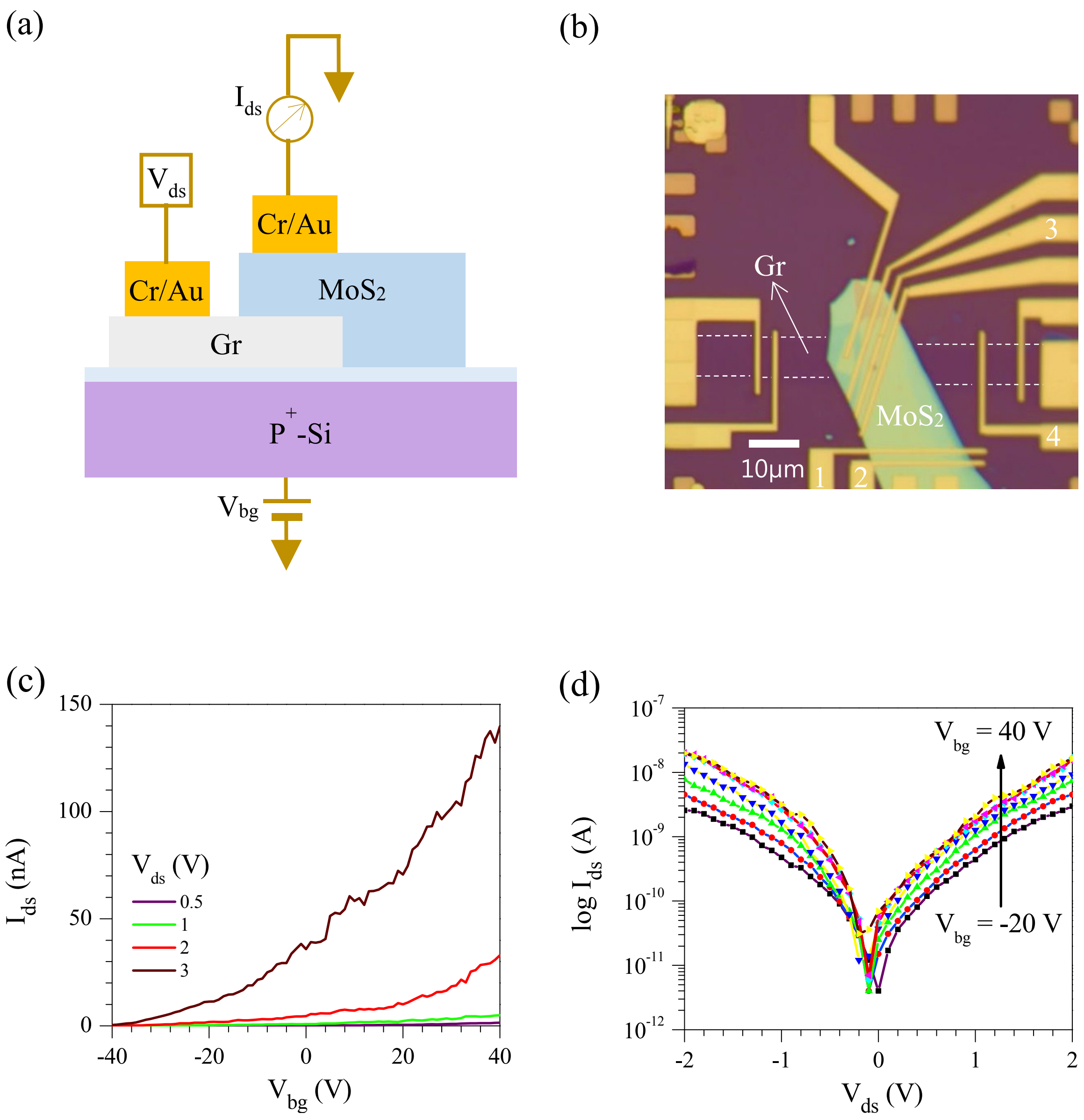

Figure S3. (a) Schematic diagram for Gr/MoS_2 device for transfer measurement. (b) Optical microscope image of Gr/MoS_2 device. (c) Transfer (I_{ds}-V_{bg}) curves of Gr/MoS_2 device at different V_{ds} measured between three and four contacts. (d) Output characteristics (I_{ds}-V_{ds}) of Gr/MoS_2 devices at various V_{bg} used for estimation of Schottky barrier height shown in Figs. 3(a) and (b). All measurement has performed at T = 300 K.

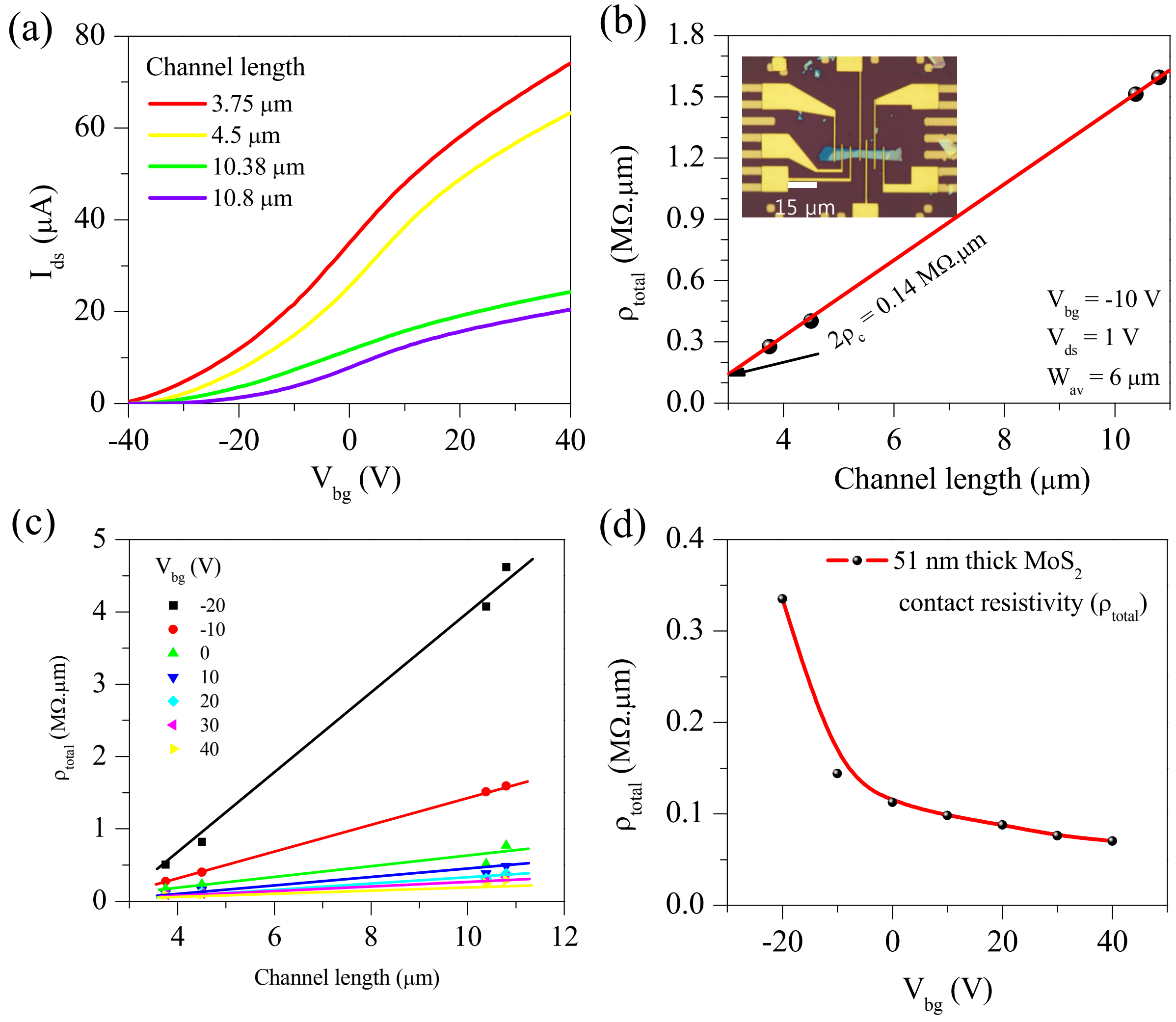

Figure S4. (a) Transfer curves (I_{ds}-V_{bg}) at V_{ds} = 1 V for different channel length in planar geometry for 51 nm-thick MoS_2 flake. (b) Total resistivity as a function of channel length based on TLM method. Extracted contact resistivity was nearly 0.14 MΩ.μm. (c) Total resistivity as a function of channel length at different V_{bg}. (d) Contact resistivity (ρ_c) as function of V_{bg}.

In order to have rough estimate of contact resistivity, we choose a MoS_2 flake with thickness of ~51 nm so, it is considered as bulk material. The contact resistivity for ~51 nm-thick flake has been measured by using transmission line method (TLM) and the results are displayed in Fig. S4. Transfer curves at different channel length for thick MoS_2 (~51 nm) flake are presented in Fig. S4(a). We choose contact resistivity instead of contact resistance in order to compare the results easily with planar resistivity of Fig. 2(d) of the main manuscript. Here we measured total resistivity (ρ_{total}) for different channel lengths at fixed V_{bg} = −10 V and then draw the straight line connecting all these resistivity points to get the intercept of *y*-axis. The value of intercept of *y*-axis then gives us the contact resistivity based on TLM method. We choose here V_{bg} = −10 V to extract contact resistivity because we measured our planar and vertical resistivity at V_{bg} = −10 V, so it would be easy to compare. We note that contact resistivity (~0.14 MΩ μm) is smaller than planar resistivity (~2.59 MΩ μm) for 51 nm-thick MoS_2 flake. Therefore, difference in the contact resistivity values from the

planar resistivity is the evidence for the existence of other factors, which contributes to the total planar resistivity as we have already discussed in our main manuscript. We also include back-gate dependent contact resistivity shown in Fig. S4(c) and (d). A large contact resistivity was found at negative V_{bg}, whereas small contact resistance was observed at positive V_{bg} due to increasing carrier channels in positive back-gate region.
